# Toll-like receptor signaling and stages of addiction

**DOI:** 10.1007/s00213-017-4560-6

**Published:** 2017-02-17

**Authors:** Fulton T. Crews, T. Jordan Walter, Leon G. Coleman, Ryan P. Vetreno

**Affiliations:** Bowles Center for Alcohol Studies, School of Medicine, University of North Carolina at Chapel Hill, Chapel Hill, NC 27599 USA

**Keywords:** HMGB1, Cytokines, miRNA let-7, Alcohol use disorder

## Abstract

**Background:**

Athina Markou and her colleagues discovered persistent changes in adult behavior following adolescent exposure to ethanol or nicotine consistent with increased risk for developing addiction. Building on Dr. Markou’s important work and that of others in the field, researchers at the Bowles Center for Alcohol Studies have found that persistent changes in behavior following adolescent stress or alcohol exposure may be linked to induction of immune signaling in brain.

**Aim:**

This study aims to illuminate the critical interrelationship of the innate immune system (e.g., toll-like receptors [TLRs], high-mobility group box 1 [HMGB1]) in the neurobiology of addiction.

**Method:**

This study reviews the relevant research regarding the relationship between the innate immune system and addiction.

**Conclusion:**

Emerging evidence indicates that TLRs in brain, particularly those on microglia, respond to endogenous innate immune agonists such as HMGB1 and microRNAs (miRNAs). Multiple TLRs, HMGB1, and miRNAs are induced in the brain by stress, alcohol, and other drugs of abuse and are increased in the postmortem human alcoholic brain. Enhanced TLR-innate immune signaling in brain leads to epigenetic modifications, alterations in synaptic plasticity, and loss of neuronal cell populations, which contribute to cognitive and emotive dysfunctions. Addiction involves progressive stages of drug binges and intoxication, withdrawal-negative affect, and ultimately compulsive drug use and abuse. Toll-like receptor signaling within cortical-limbic circuits is modified by alcohol and stress in a manner consistent with promoting progression through the stages of addiction.

## Introduction

Several studies have found lasting changes in brain that contribute to the progression through the stages of addiction (Koob and Volkow 2010). Emerging findings suggest that signaling through known innate immune signaling systems in brain contributes to mounting negative affect and may underlie the neurobiological similarities of depression and addiction (Markou et al. 1998). Athina Markou and her colleagues discovered persistent changes in adult behavior following adolescent exposure to ethanol or nicotine consistent with increased risk for developing addiction. For example, adolescent alcohol exposure was shown to increase high-reward choices even when the probability of reward outcome was unlikely (i.e., rats exposed to ethanol during adolescence led to increased risky decision-making in adulthood; Boutros et al. 2014, 2015). Risky choice preference was found to correlate with the loss of cholinergic neurons in the basal forebrain (Boutros et al. 2014). Adolescent ethanol exposure was also found to increase adult central nucleus of the amygdala corticotropin-releasing factor (CRF) messenger RNA (mRNA) 40 days following the last ethanol dose (i.e., a persistent change in amygdalar CRF levels; Boutros et al. 2016). Similarly, 20 days after the last ethanol exposure, adolescent, but not adult rats, were found to have a long-lasting decrease in time spent in the open arms of a radial arm maze, consistent with deficits in risk-reward integration (Risher et al. 2013). Further, this study found a lasting change in sensitivity to acute ethanol disruption of working memory (Risher et al. 2013), and another study using delayed discounting found that acute ethanol weeks after adolescent exposure altered impulsivity (Mejia-Toiber et al. 2014). These studies are consistent with findings indicating that adolescent ethanol exposure alters adult reward responses to ethanol (Boutros et al. 2014). Taken together, these findings and others are consistent with adolescent ethanol causing persistent changes in brain that increase risk for addiction in adulthood (Crews et al. 2016). These persistent changes in behavior following adolescent stress or alcohol exposure may be linked to induction of immune signaling in brain.

**Fig. 1:**
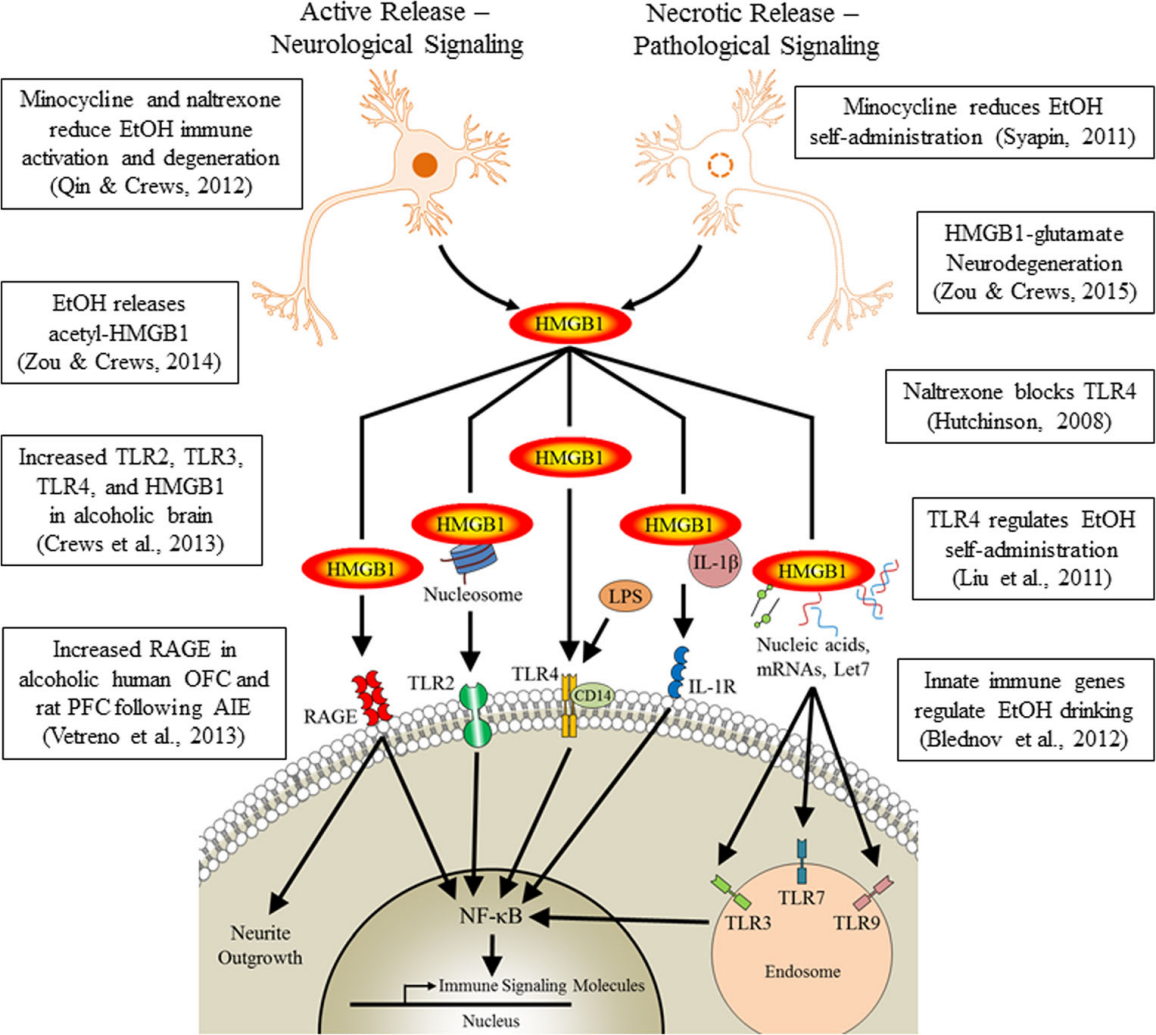
High-mobility group box 1 (HMGB1) signaling involvement in addiction neuropathology. HMGB1 is actively and/or passively released from neurons and other cells leading to the activation of multiple innate immune signaling pathways. Extracellularly, HMGB1 can directly interact with toll-like receptors (TLRs) or form complexes with various ligands to enhance immune responses at various pattern recognition receptors (PPRs). In addition, HMGB1 can form complexes with nucleic acids and microRNAs (miRNAs; e.g., let-7) that are endocytosed into the cell-activating intracellular TLRs. Activation of TLRs and other PRRs leads to activation of the transcription factor nuclear factor kappa-light-chain-enhancer of activated B cells (NF-κB) and subsequent induction of proinflammatory cytokines and oxidases that are released into the extracellular space. Evidence implicates these cascades in contributing to the progression through the stages of addiction. *LPS* lipopolysaccharide, *RAGE* receptor for advanced glycation end-products, *IL-1β* interleukin-1beta

**Fig. 2:**
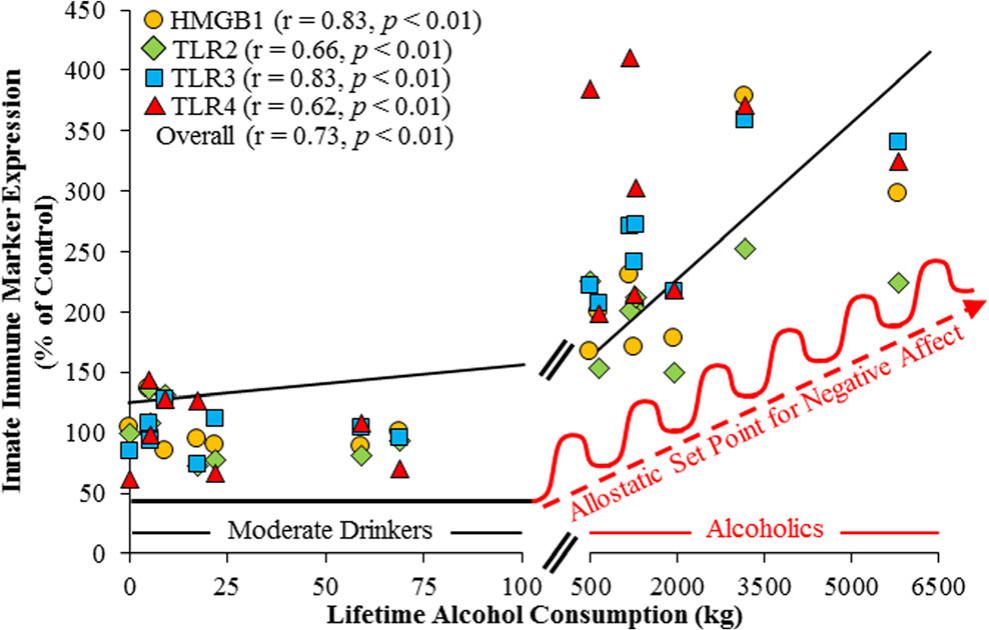
Lifetime alcohol consumption and HMGB1-TLR expression in the human postmortem orbitofrontal cortex. Expression of toll-like receptors (TLRs) 2, 3, and 4 and the TLR endogenous agonist high-mobility group box 1 (HMGB1) are positively correlated with lifetime alcohol consumption (kg) (Crews et al. 2013). Moderate drinking controls are clustered along the *left of the graph* due to low lifetime levels of alcohol consumption and concomitant low levels of TLR-HMGB1 expression. Alcoholic subjects all consumed more alcohol than moderate drinking controls but show a 10-fold variation in lifetime alcohol consumption. Note that the *x* axis is broken to allow for visualization of the moderate drinking control data. Repeated cycles of binge drinking are hypothesized to induce a progressive and persistent shift in the allostatic set point for negative affect that contributes to the neurobiology of addiction (Koob and Le Moal 2005). Depicted are correlations for individual TLRs and HMGB1 and overall grouped correlations with lifetime alcohol consumption

Over the past decade, the innate immune system has emerged as a critical component in the development of alcohol use disorders and other addictions. Microglia, the resident macrophage-like innate immune cells of the central nervous system, have only recently been discovered to play a critical role in brain homeostasis with key roles in neuronal differentiation, synapse formation, and neurocircuitry. It was only recently discovered that on embryonic day 8 in mice, mesodermal progenitor yolk sac cells migrate to brain to form microglia that persist throughout life as unique brain cells, distinct from the neuro-ectodermal cells that form neurons and other glia (e.g., astrocytes and oligodendrocytes) (Ginhoux et al. 2010, 2013). Originating from the mesoderm, microglia express a multitude of innate immune signaling receptors and molecules. One microglia-associated receptor system, the toll-like receptor (TLR) superfamily (i.e., interleukin-1 receptor/toll-like receptor), mounts proinflammatory responses to pathogens by sensing large molecules containing lipids, sugars, protein, and nucleic acid components. Initial studies suggested that only microglia are involved in TLR signaling, but more recent studies suggest that all brain cell types are involved in this signaling (Crews and Vetreno 2016). Toll-like receptors activate signaling cascades that converge on nuclear factor kappa-light-chain-enhancer of activated B cells (NF-κB), a key innate immune nuclear transcription factor that promotes expression of proinflammatory cytokines, including tumor necrosis factor α (TNFα), interleukin-1β (IL-1β), IL-6, and monocyte chemoattractant protein-1 (MCP-1). Recent studies in brain, which is normally a sterile environment, indicate that endogenous TLR agonists may comprise an important signaling pathway between microglia, other glia, and neurons. The ubiquitously expressed nuclear protein, high-mobility group box 1 (HMGB1), is a cytokine-like molecule expressed in all cell types that upon release can directly activate TLRs and enhance the response of other innate immune signaling molecules at their respective receptors (see Fig. 1). Human and preclinical animal studies find that stress (Frank et al. 2015), alcohol (Crews and Vetreno 2014), and other drugs of abuse (Hutchinson et al. 2010; Northcutt et al. 2015) increase expression of TLRs and other innate immune signaling molecules (e.g., HMGB1) in brain. Induction of TLRs and HMGB1, an endogenous TLR agonist, leads to activation of positive loops of amplification that result in a progressive and persistent increase in TLR and HMGB1 signaling. For example, expression of HMGB1 and multiple TLRs in the postmortem human orbitofrontal cortex are correlated with lifetime alcohol consumption in alcoholic and moderate drinking controls (Crews et al. 2013). Cycles of binge drinking may sensitize the brain to promote drinking behavior (see Fig. 2). Similarly, mechanistic preclinical studies of epilepsy reveal that neuronal activation causes the rapid release of HMGB1 and IL-1β that occur prior to seizure onset resulting in neuronal sensitization to excitation due to alterations in excitatory receptor expression as well as lowered seizure threshold leading to increased seizure frequency and severity with each event (i.e., “kindling”; Maroso et al. 2011). While the brain regions and neural circuits underlying addiction and epilepsy differ, stress and drug abuse may share a common mechanism of neurocircuitry sensitization through increased HMGB1 and TLR signaling.

Preclinical models reveal that ethanol increases the expression of HMGB1 and TLRs in the brain that persists through long periods of abstinence (Vetreno and Crews 2012; Vetreno et al. 2013). Human studies find increased expression of microglial markers and cytokines as well as HMGB1 and TLRs in the postmortem alcoholic brain (Crews et al. 2013; He and Crews 2008; Zou and Crews 2012) with the latter two correlating with lifetime alcohol consumption (Crews et al. 2013). Repeated stress and cycles of alcohol and drug abuse sensitize microglia consistent with the hypothesis that induction of innate immune signaling pathways contributes to the progressive increase in craving, mood dysfunction, and cognitive impairments observed in addiction. Recent discoveries indicate that ethanol leads to the induction of multiple TLRs as well as endogenous TLR agonists in brain. While poorly understood at present, studies suggest that activation and escalated signaling of this system lead to a progressive loss of behavioral control, increased impulsivity and anxiety, and negative affect and craving coupled with increasing ventral striatal responses to promote reward-seeking behaviors and increase the risk of developing alcohol use disorders.

## The innate immune system

**Fig. 3:**
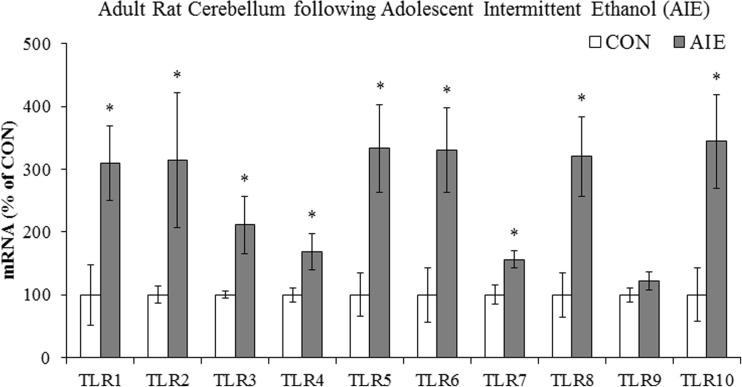
Adolescent intermittent ethanol (AIE) treatment upregulates expression of toll-like receptors (TLRs) in the adult cerebellum. From postnatal day (P)25 to P55, AIE-treated male Wistar rats received a single daily intragastric dose of ethanol (5.0 g/kg, 20% ethanol, *w*/*v*) on a 2-day on/off schedule, and CON subjects received comparable volumes of water as described previously (Vetreno et al. 2016). Following a 25-day abstinence period, cerebellar tissue samples were collected on P80. Tissue samples were processed for qPCR and TLR mRNAs assessed as previously described (Vetreno and Crews 2012). Data are presented as mean ± SEM. *p < 0.05

While early studies characterized innate immune receptors based on their response to specific pathogens (e.g., bacteria and viruses), more recent “sterile inflammation” studies in brain led to the identification of these receptors as pattern recognition receptors (PRRs). Pattern recognition receptors have revolutionized our understanding of innate immune system signaling in brain. These receptors recognize and respond not only to specific molecular patterns present on foreign (exogenous) pathogens (i.e., pathogen-associated molecular patterns [PAMPS]) but also to endogenous signaling molecules associated with cell damage, degeneration, or stress (i.e., danger-associated molecular patterns [DAMPs]) (Bianchi 2007). To date, five classes of PRRs have been characterized: (1) TLRs, (2) C-type lectin receptors, (3) nucleotide-binding oligomerization domain-like receptors (NOD-like receptors), (4) RIG-I-like receptors, and (5) AIM2-like receptors (Brubaker et al. 2015). Although all of these receptors are important for host defense, the TLR family of PRRs has most thoroughly been characterized using inflammagens, such as endotoxin/lipopolysaccharide at TLR4 and viral RNA at TLR3 and TLR7. Toll-like receptors share an extracellular N-terminal leucine-rich repeat sequence and an intracellular toll/interleukin-1 receptor/resistance motif (TIR; Takeuchi and Akira 2010). Currently, 10 TLRs have been identified in humans and 12 TLRs have been identified in mice (Brubaker et al. 2015). All of these PRRs recognize a variety of PAMPs, including bacterial endotoxin and viral RNA as well as DAMPs such as mammalian HMGB1, microRNAs, and heat shock proteins (Vabulas et al. 2002). Interestingly, DAMP activation of TLRs has been identified as a contributing factor in several non-infectious neurological disorders, including alcoholism. For instance, expressions of TLR2, TLR3, and TLR4 as well as the PRR receptor for advanced glycation end-products (RAGE) and the TLR/RAGE endogenous agonist HMGB1 are each increased in the postmortem human alcoholic orbitofrontal cortex (Crews et al. 2013; Vetreno et al. 2013). Preclinical models of adolescent binge ethanol treatment find long-term upregulation of TLR3, TLR4, RAGE, and proinflammatory cytokines in the adult frontal cortex (Vetreno and Crews 2012) as well as several TLRs (TLR1–TLR10 except for TLR9) in the adult cerebellum (see Fig. 3). Immune signaling through TLRs and other immune signaling receptors induce additional TLRs and other innate immune signaling agonists and their respective receptors, which further induce immune signals, creating positive loops of activation that likely amplify and contribute to the persistence of drug-induced immune signaling. Similarly, other studies report that chronic ethanol exposure increases expression of TLR2 in the mouse brain (Fernandez-Lizarbe et al. 2013; Oak et al. 2006). Thus, both human and animal studies find that alcohol upregulates the expression of multiple TLRs.

In order for these innate immune signaling molecules to remain elevated for extended periods of abstinence, signaling amplification of these signaling molecules likely occurs. Ethanol causes activation of the transcription factor NF-κB, leading to increased PRR expression, cytokine induction, and DAMP release. This signaling system leads to amplification of the innate immune response in brain through an autocrine and paracrine process such that secreted cytokines, DAMPs, and microRNAs activate receptors on the cells of origin as well as surrounding cells. In addition, ethanol causes generation of reactive oxygen species that further activates NF-κB transcription of cytokines and DAMPs in an autocrine and paracrine fashion (Pietri et al. 2005; Qin and Crews 2012b; Thakur et al. 2007). Thus, the gradual induction of inflammatory processes that accompanies repeated binge-drinking episodes, particularly during adolescence, leads to a progressive and persistent activation of the innate immune system and might underlie the transition from alcohol abuse to alcoholism.

As alluded to above, TLR signaling pathways are quite complex. Following recognition of a ligand by a specific TLR, the adaptor protein TIRAP/MyD88, which interacts with all of the TLRs except TLR3, initiates intracellular signaling cascades leading to downstream activation of IL-1 receptor-associated kinases (IRAKs) and TNF receptor-associated factor 6 (TRAF6), which cause IκB and MAPK activation. Activation of IκB and MAPK in turn leads to downstream activation of the transcription factor NF-κB and subsequent cytokine induction (Crews et al. 2013; Lippai et al. 2013b). NF-κB is a critical modulator of innate immune function, and exposure to stress and drugs of abuse result in NF-κB activation. For instance, psychosocial stress induces NF-κB activation in human blood mononuclear cells (Bierhaus et al. 2003). In rodents, restraint stress increases expressions of NF-κB, proinflammatory cytokines, and the proinflammatory oxidase cyclooxygenase-2 (COX-2) in brain (Madrigal et al. 2002, 2003). Ethanol also activates NF-κB in rat and mouse brain (Qin and Crews 2012b; Ward et al. 1996), and NF-κB target genes are upregulated in the postmortem human alcohol prefrontal cortex (Okvist et al. 2007). Cocaine exposure also leads to activation of NF-κB in the nucleus accumbens and is required for the establishment of cocaine-induced, conditioned place preference (Ang et al. 2001a; Russo et al. 2009). Thus, both stress and drugs of abuse lead to activation of the innate immune transcription factor NF-κB in the brain that might contribute to innate immune induction.

## Innate immunity, TLR signaling, and addiction

**Fig. 4:**
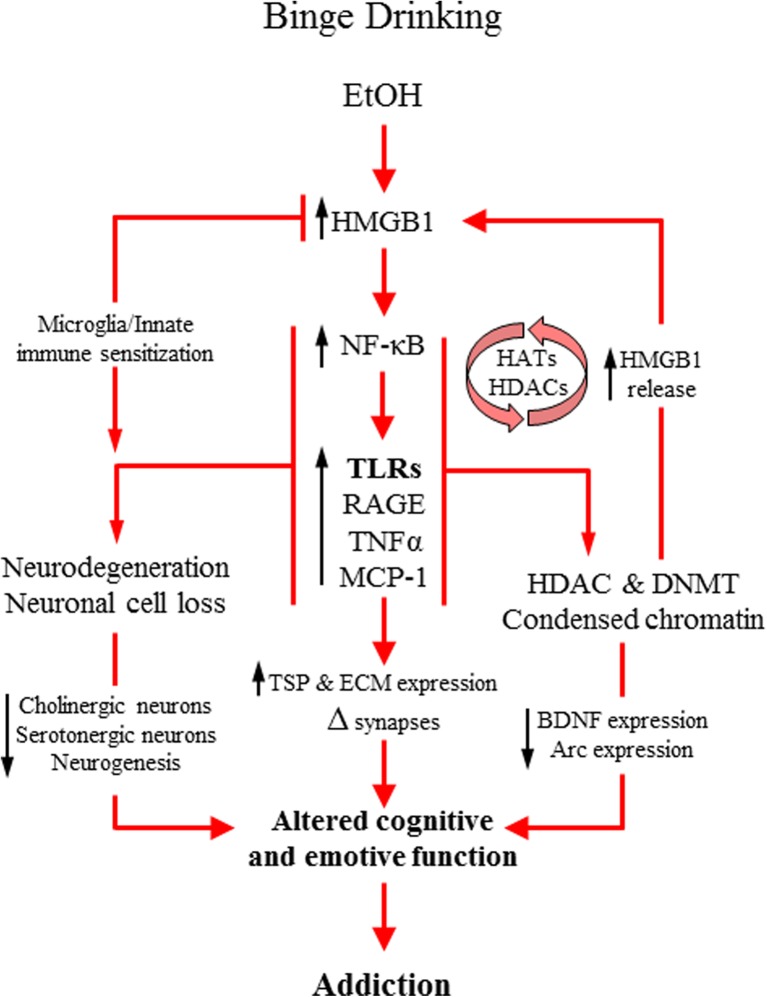
Simplified schematic depicting mechanisms of alcohol-induced alterations to brain plasticity. Alcohol binge drinking and abuse increases expression of the endogenous innate immune receptor agonist high-mobility group box 1 (HMGB1) leading to activation of the transcription factor nuclear factor kappa-light-chain-enhancer of activated B cells (NF-κB) and subsequent induction of proinflammatory cytokines and oxidases (Crews et al. 2013; Qin and Crews 2012a; Vetreno and Crews 2012, 2015; Vetreno et al. 2016). This also leads to increased expression of toll-like receptors (TLRs) and the receptor for advanced glycation end-products (RAGE) (Crews et al. 2013; Vetreno and Crews 2012, 2015; Vetreno et al. 2013), which are receptors for HMGB1. Activation of this signaling pathways leads to the establishment of positive loops of amplification that persist during abstinence from alcohol (Crews and Vetreno 2016) and alter synapse formation through increased expression of thrombospondins (Risher et al. 2015) and alterations in extracellular matrix proteins (Coleman et al. 2014). Concomitantly, innate immune induction causes epigenetic modifications that induce further release of HMGB1 (Zou and Crews 2014) that contribute to positive loops of amplification as well as alterations in synaptic plasticity molecules (Pandey et al. 2015; Sakharkar et al. 2016) that contribute to alterations in cognitive and emotive functioning. Innate immune driven epigenetic modifications might also lead to microglial alterations resulting in the reprogramming of the innate immune system. Innate immune induction also causes neurodegeneration (Braun and Crews 2000; Crews et al. 2000) and the loss of neuron-specific cell populations (Boutros et al. 2015; Vetreno et al. 2014; Vetreno and Crews 2015; Vetreno et al. 2016) that might also contribute to the dysfunctional cognitive and affective states observed in addiction (Crews and Vetreno 2016)

There is evidence consistent with the hypothesis that stress- and drug-induced innate immune upregulation inactivates the prefrontal cortex and sensitizes limbic circuitry. This suggests that the progressive and persistent increase of HMGB1-TLR signaling occurring with each cycle of stress and drug abuse is the mechanism underlying addiction (Crews et al. 2011; Vetreno and Crews 2014). Induction of innate immune signaling is known to cause cognitive and emotive dysfunctions in both preclinical (Dantzer et al. 2008; Hanke and Kielian 2011; Okun et al. 2010; Yirmiya and Goshen 2011) and human studies (Brites and Fernandes 2015; Critchley and Harrison 2013; Harrison et al. 2015; Rea et al. 2016). Alcohol and other drugs of abuse promote innate immune induction (Crews et al. 2016; He and Crews 2008; Qin et al. 2008) that is associated with alterations in executive function, reinforcement, and affective processes that promote alcohol abuse and addiction (Vetreno and Crews 2014). Indeed, ethanol induction of innate immune activation and TLR signaling contribute to brain alterations and the development of addiction-like behaviors (Liu et al. 2011b; Montesinos et al. 2016; Pascual et al. 2011, 2015). For instance, Guerri and colleagues (Pascual et al. 2011) found that binge ethanol-induced innate immune activation in mice impairs novel object recognition memory and increases anxiety-like behavior, an effect that is not found in TLR4 knockout mice. Interestingly, the observed behavioral impairments in this study were accompanied by reduced H3 and H4 histone acetylation as well as diminished histone acetyltransferase activity in the frontal cortex, striatum, and hippocampus, which was also not observed in TLR4 knockout mice. The finding that mice lacking TLR4 do not evidence ethanol-induced changes in epigenetic markers suggests that TLR-innate immune signaling contributes to drug-induced epigenetic changes leading to alterations in neurotransmitter signaling related to addiction. Similarly, adolescent binge ethanol exposure increases both ethanol preference and cocaine-conditioned place preference in young adult rodents, which was not observed in TLR4 knockout mice (Montesinos et al. 2016) or adult rats following treatment with trichostatin A, a histone deacetylase inhibitor (Pandey et al. 2015). These findings suggest that TLRs, specifically TLR4 in these experiments, are required for chronic ethanol-induced behavioral and histone acetylation changes in brain. TLR-innate immune induction appears to drive epigenetic and behavioral modifications that likely play a role in the dysfunctional cognitive, emotive, and reinforcement processes observed in addiction, since mice lacking TLR4 do not show these ethanol responses (Montesinos et al. 2016). Although the epigenetic mechanism underlying these effects is unclear, the data suggest that these changes might be due in part to increased innate immunity and diminished neurotropic expression that leads to alterations in synaptic plasticity (see Fig. 4). Indeed, adolescent intermittent ethanol (AIE) -induced activation of astrocytes, which are an important component of the innate immune system (Farina et al. 2007), is associated with increased expression of thrombospondins, which regulate synapse formation (Christopherson et al. 2005) in vitro and in the adult hippocampus (Risher et al. 2015). Thus, alcohol exposure may increase neuroplasticity in brain regions associated with addiction. An additional inflammatory pathway mediated by RAGE might also contribute to addiction and the cognitive impairments associated with alcoholism. Studies in the postmortem human alcoholic brain and AIE model find increased expression of RAGE in the adult prefrontal cortex (Vetreno et al. 2013), and this receptor has been implicated in memory impairments associated with Alzheimer’s disease (Arancio et al. 2004; Fang et al. 2010; Maczurek et al. 2008; Wilson et al. 2009). Together, these data connect TLRs, RAGE, HMGB1, and other innate immune signaling molecules with epigenetic modifications to drug- and stress-induced changes in neurobiology that are related to addiction.

Although TLRs are capable of sensing and responding to a multitude of ligands, HMGB1 has been identified as a critical mediator of innate immune induction in alcohol abuse. HMGB1 is a nuclear protein involved in chromatin stabilization and acts as a chaperone for various transcription factors (Thomas and Stott 2012). Following extracellular release, HMGB1 binds to TLR4 or RAGE depending on its redox state causing induction of innate immune responses (Janko et al. 2014). Specifically, the Cys106 thiol/Cys23-Cys45 disulfide form of HMGB1 acts at TLR4 while the fully reduced form of HMGB1 is active at the RAGE receptor (Venereau et al. 2012; Yang et al. 2012). Ethanol has been found to cause nuclear translocation of HMGB1 through an epigenetic process leading to cellular release in ex vivo slice culture (Zou and Crews 2014). Expression of HMGB1 is increased in the prefrontal cortex of postmortem human alcoholics and adult rats following adolescent binge ethanol exposure (Crews et al. 2013; Vetreno and Crews 2012; Vetreno et al. 2013). In addition to direct receptor binding, extracellular HMGB1 can form heterodimers with a number of molecules, including lipoglycans, endotoxin, cytokines (e.g., IL-1β), and nucleic acids (e.g., microRNAs) (Bianchi 2009; Hreggvidsdottir et al. 2009), resulting in increased potency of immune responses. Further, HMGB1 is necessary for the induction of immune responses by endosome nucleic acid-binding TLRs (i.e., TLR3, TLR7, and TLR9) (Yanai et al. 2009), although the mechanism remains to be fully elucidated. Thus, alcohol-induced release of HMGB1 might activate multiple TLRs contributing to innate immune induction in brain.

In addition to HMGB1-TLR signaling pathways, ethanol also modulates innate immune function through the release of microRNAs (miRNAs), which are small, non-coding RNAs (∼22 nucleotides) that regulate innate immunity (Thounaojam et al. 2013). At present, miRNAs are thought to regulate innate immunity through two processes that involve (1) the stabilization of target mRNAs in the cytosol (Ambros 2004; Bartel 2004; Czech and Hannon 2011) and (2) extracellular release of miRNAs (Ambros 2004; Bartel 2004; Czech and Hannon 2011). Previous studies have found that upregulation of miRNAs is associated with psychiatric disorders (O’Connor et al. 2016) and addiction (Dreyer 2010), although the precise mechanism and impact on innate immune signaling are uncertain. In the extracellular milieu, miRNAs are either bound to lipoprotein chaperones or encapsulated in extracellular vesicles (EVs) that can be endocytosed by neighboring cells, thereby modulating their function. Ethanol exposure has been found to alter miRNA expression in the brain. Encapsulated miRNAs from ethanol-treated monocytes were found to modulate the activation state of naïve monocytes (Saha et al. 2016). Further, alcohol alters the expression profile of multiple miRNAs in the frontal cortex of postmortem human alcoholics and mice (Lewohl et al. 2011; Nunez and Mayfield 2012; Nunez et al. 2013). Interestingly, the miRNAs let-7 and miR-21 were recently identified as endogenous endosomal TLR7 agonists (Lehmann et al. 2012; Yelamanchili et al. 2015), and our laboratory found that ethanol potentiates TLR7-mediated innate immune induction through the release of let-7 in microvesicles (Coleman et al. 2017). The miRNA miR-155 is also found in vesicles and promotes TLR4-associated innate immune responses following chronic ethanol exposure (Lippai et al. 2013a). Although poorly understood at present, the release of miRNAs is an additional mechanism through which ethanol activates TLRs and the innate immune system, thereby contributing to the stages of addiction.

Animal studies provide compelling evidence that the innate immune system modulates alcohol consumptive behaviors. Analysis of genetically paired rats and mice reveals that high ethanol-drinking animals evidenced higher levels of NF-κB and other proinflammatory genes relative to their low-drinking litter mates (Mulligan et al. 2006). Indeed, expression of β2-microglobulin, a NF-κB target gene involved in immune signaling (Pahl 1999), was elevated in high ethanol-preferring brain transcriptomes (Mulligan et al. 2006). Studies in transgenic mouse lines provide further support for the hypothesis that the innate immune system regulates ethanol-drinking behaviors. Across multiple ethanol-drinking paradigms, transgenic mice with deletion of a single immune gene, such as CD14 and IL-6, consumed significantly less ethanol than matched wild-type controls (Blednov et al. 2005b, 2012). Further, Blednov and colleagues (Blednov et al. 2011) found that a single dose of LPS, the prototypical TLR4 agonist, produced a delayed but long-lasting increase of ethanol self-administration in mice. Similarly, site-specific microinjections of a GABA_A_α2 small interfering RNA (siRNA) vector into the central nucleus of the amygdala of alcohol-preferring rats diminished binge drinking, which was associated with reduced expression of TLR4 (Liu et al. 2011a). As GABA_A_α2 receptors are located on neurons, these data suggest that involvement of TLR4 in binge drinking is at least partially mediated by neurons. Indeed, both postmortem human and rodent studies find that TLR2–TLR4, RAGE, and HMGB1 are expressed on neurons (Crews et al. 2013; Vetreno and Crews 2012; Vetreno et al. 2013). Thus, there is accumulating data for a neuromodulatory role of the innate immune system in driving alcohol preference and consumption.

**Fig. 5:**
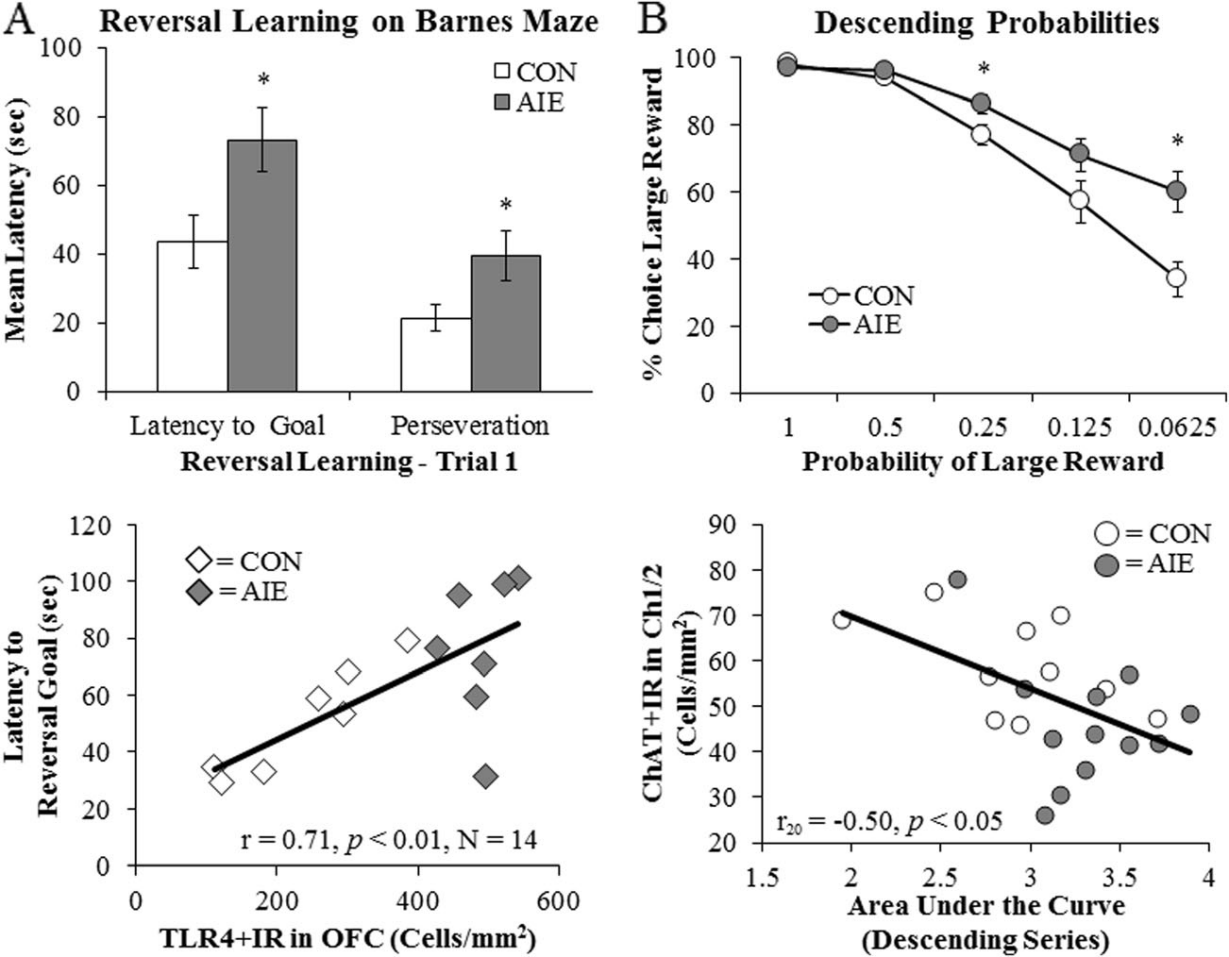
Adolescent intermittent ethanol (AIE) treatment impairs reversal learning and increases risky decision-making in adulthood. **a** From postnatal day (P)25 to P55, AIE-treated male Wistar rats received a single daily intragastric (i.g.) dose of ethanol (5.0 g/kg, 20% ethanol, *w*/*v*) on a 2-day on/off schedule, and CON subjects received comparable volumes of water as previously described (Vetreno and Crews 2012). Following a 25-day abstinence period, spatial and reversal learning were assessed on the Barnes maze. While AIE treatment did not impair spatial learning, reversal learning, which provides a measure of behavioral flexibility, was impaired in adulthood as evidenced by increased latency to the reversal goal and increased perseveration. Following behavioral testing, brain tissue was collected and tissue stained for innate immune markers. Expression of toll-like receptor 4 (TLR4) was positive correlated with latency to the reversal goal, indicating that innate immune induction by AIE contributes to deficits in adult deficits in behavioral flexibility. Adopted from Vetreno and Crews (2012). Data are presented as mean ± SEM. *p < 0.05. **b** From P28 to P53, male Wistar rats received three daily i.g. doses of ethanol (5.0 g/kg, 25% ethanol, *w*/*v*) on a 2-day on/off schedule, and CON subjects received comparable volumes of water. Following an abstinent period on P63, training and testing were performed on the probability-discounting task. Prior AIE treatment significantly increased risky responses at reducing reward probabilities, relative to CON subjects. Expression of choline acetyltransferase (ChAT), a marker of cholinergic neurons, was reduced in the basal forebrain of adult AIE-treated animals that was negatively correlated with probability-discounting behavior. Adopted from Markou and colleagues (Boutros et al. 2015). Data are presented as mean ± SEM. *p < 0.05

The frontal cortex mediates executive functions, including motivation, planning and goal setting, and behavioral flexibility. In non-alcoholic social drinking humans, the heaviest binge drinkers report more negative mood and perform worse on executive functioning tasks (Townshend and Duka 2003; Weissenborn and Duka 2003), suggesting that diminished frontal cortical function occurs with binge drinking. In astrocytes, ethanol causes NF-κB transcription and downstream induction of proinflammatory innate immune genes (Pascual et al. 2007; Zou and Crews 2006, 2010) as well as a loss of astrocyte glutamate transport (Zou and Crews 2005). Elevated levels of extracellular glutamate result in increased neuronal excitation, microglial activation, and excitotoxicity (Ward et al. 2009; Zou and Crews 2006). In the frontal cortex, ethanol-induced glutamate excitotoxicity results in increased caspase-3 and COX-2 expressions that require TLR4 signaling (Alfonso-Loeches et al. 2010; Knapp and Crews 1999). Similar glutamate hyperexcitability also occurs in the stimulant-addicted brain (Reissner and Kalivas 2010). Activation of the innate immune system reduces glutamate transporters, causing hyperexcitability that reduces frontal cortical executive function, thereby contributing to the neurobiology of addiction (Crews et al. 2006a, 2011). Indeed, frontal cortical dysfunction is common in the alcoholic brain (Crews and Boettiger 2009) and is manifest in impulsivity and impaired behavioral flexibility. In preclinical models, reversal learning tasks are used to assess frontal cortical function. Reversal learning provides a measure of behavioral flexibility, which refers to the animals’ ability to adopt a new behavioral response as a consequence of a shift in task demands. Typically, impairments in reversal learning are associated with increased perseveration of previously learned behaviors or the inability to break previously learned responses. Studies from our laboratory have consistently revealed persistent reversal learning deficits in adult rats following binge ethanol exposure (Obernier et al. 2002c) as well as in adult rats and mice following AIE treatments (Coleman et al. 2011; Vetreno and Crews 2012). Interestingly, we found that measures of reversal learning and perseveration were correlated with expression of TLRs, RAGE, and HMGB1 in the adult prefrontal cortex following AIE (Vetreno and Crews 2012; Vetreno et al. 2013) (see Fig. 5a). Additionally, deficits in reversal learning are observed in rats that self-administer cocaine or are exposed to passive cocaine injections (Calu et al. 2007; Schoenbaum et al. 2004), suggesting that behavioral flexibility deficits are common across addiction. Involvement of the frontal cortex in reversal learning is further supported by lesion-induced reversal learning deficits that are similar in nature to chronic drug abuse-induced dysfunction (Schoenbaum et al. 2006). Adolescent binge drinking is associated with increased impulsivity (White et al. 2011) and risky decision-making (Goudriaan et al. 2007). In preclinical models, risk-based decision-making is assessed using the probability-discounting task, in which rats must select either a small reward (single food pellet) that is always delivered (i.e., safe reward) or a large reward (four food pellets) that is delivered with decreasing frequency (i.e., risky choice) across trials. Elegant studies by Markou and colleagues (Boutros et al. 2015) investigated risk-based decision-making in rats using a probability-discounting task, in which rats must select either a small reward (single food pellet) that is always delivered (i.e., safe reward) or a large reward (four food pellets) that is delivered with decreased frequency (i.e., risky choice) across trials. Control animals switched from the high-reward level when more responses were required, reducing pellets, whereas AIE-treated animals demonstrated an increased preference for the risky large reward, even when many more responses were required. This suggests that AIE increased adult risky decisions (Boutros et al. 2015). Interestingly, increased risky decision-making was negatively correlated with expression of choline acetyltransferase, a marker of cholinergic neurons, in the basal forebrain, also reduced by AIE (see Fig. 5b). Basal forebrain cholinergic neurons provide acetylcholine inputs to the frontal cortex, which are important modulators of frontal cortical executive function (Baxter and Chiba 1999) and impulsivity (Sarter and Paolone 2011). Treatment of rats with LPS, the TLR4 agonist, causes a reduction of cholinergic neurons similar to the effects of AIE on cholinergic populations in adulthood. These data implicate innate immune activation through TLR4 in the loss of specific neuron populations that might contribute to behavioral deficits commonly observed in addiction. Taken together, considerable evidence is emerging that supports a role for HMGB1/TLR4 signaling, innate immune gene induction, and epigenetic alterations as culminating in the neurobiology of addiction (Crews et al. 2011; Cui et al. 2015; Vetreno and Crews 2014).

## Addiction, stress, and innate immune signaling

Cycles of exposure to drugs of abuse and stress interact, contributing to a progressive sequence of stages leading to addiction (Volkow et al. 2016). Both drugs of abuse and stress activate innate immune proinflammatory responses. Stress-induced innate immune responses can have beneficial or maladaptive consequences on brain function. Physical stressors, such as injury and infection, induce proinflammatory cytokines and other signaling molecules that trigger adaptive behavioral changes known as “sickness behavior.” Sickness behaviors, which include social withdrawal, decreased activity, somnolence/sleepiness, and anhedonia, help facilitate energy conservation and recovery from illness (Dantzer et al. 2008). Interestingly, even psychological stressors (e.g., social stress) can induce inflammatory responses (Steptoe et al. 2007). The adaptive response of sickness behavior to acute stress and inflammation is presumably beneficial; however, prolonged or chronic exposure to stress and inflammation can become maladaptive, leading to neuropsychiatric disease, such as depression. Indeed, the similarities between sickness behavior and depression are well known (Maes et al. 2012). Chronic innate immune activation following prolonged alcohol and drug abuse likely results in similar psychopathologies. Thus, persistent innate immune induction caused by chronic alcoholism might lead to maladaptive sickness behavior that manifests as cognitive and emotive dysfunctions.

**Fig. 6:**
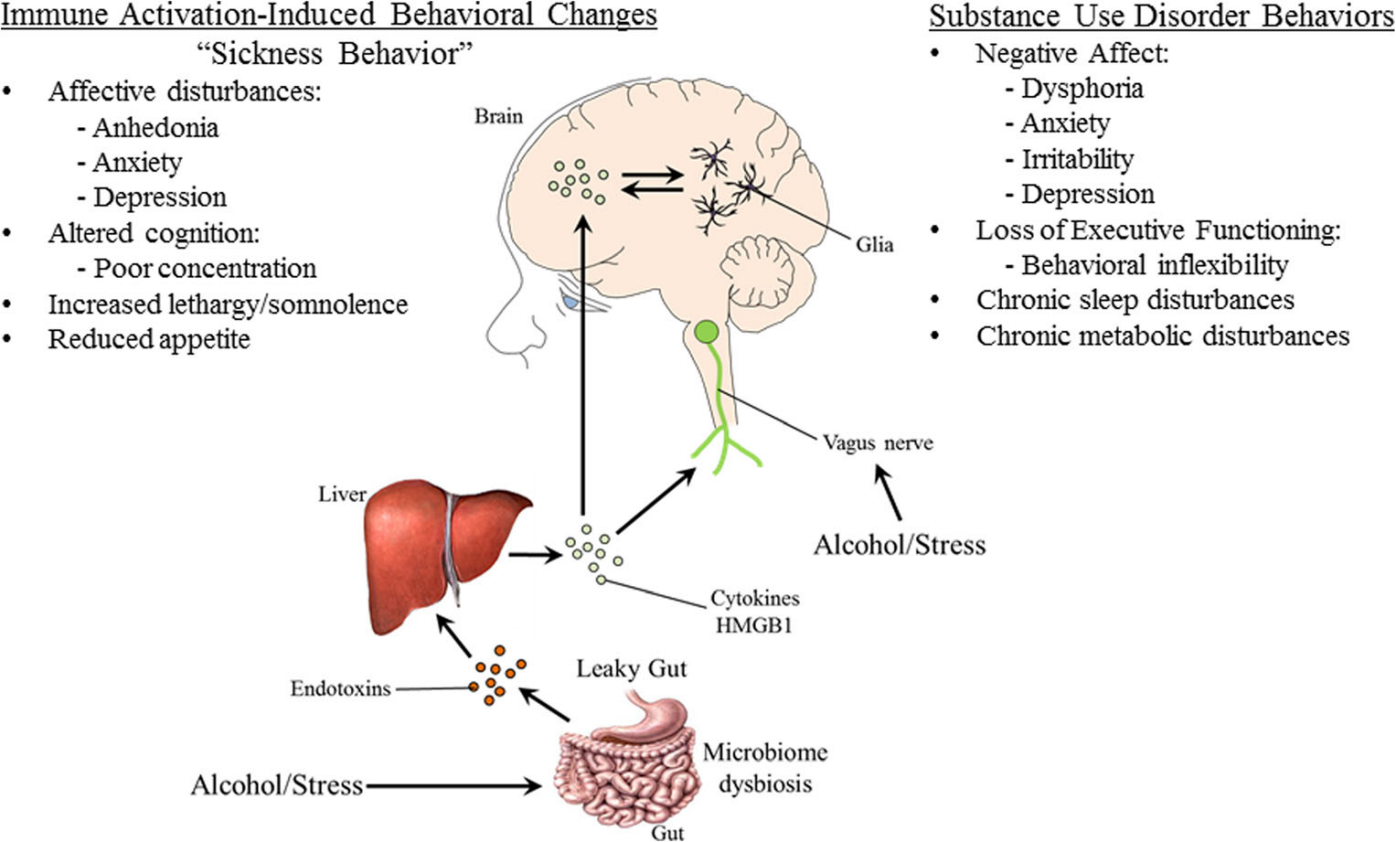
Mechanisms of stress- and ethanol-induced innate immune activation. Alcohol and stress activate the peripheral and central immune systems in multiple ways. Alcohol and stress cause microbiome dysbiosis and disrupt gut tight junctions, leading to permeability of the gut and release of bacteria and endotoxin that enter portal circulation-inducing inflammatory responses in the liver. The consequent release of inflammatory cytokines from the liver activates the innate immune system in brain through direct transport via cytokine receptors and activation of the vagus nerve. Stress and alcohol exposure leads to innate immune activation in brain that induces sickness-like behaviors that contribute to the stages of addiction. These pathways of ethanol- and stress-induced activation of HMGB1-LTR signaling likely contribute to the persistent and progressive stages of addiction

Recent studies find that stress and alcohol contribute to inflammation through a similar mechanism of increased intestinal permeability. Stress causes intestinal permeability and bacterial translocation from the gut lumen (Garate et al. 2013). In response to the leaked bacteria, the peripheral immune system mounts a proinflammatory immune response. At binge levels, alcohol (i.e., >2 g/kg; Ferrier et al. 2006) also causes disruption of gut tight junctions, allowing for the translocation of gut bacteria into the periphery (Adachi et al. 1995). The bacteria enter portal circulation leading to inflammation of the liver and release of proinflammatory cytokines into systemic circulation (Crews and Vetreno 2014; Mayfield et al. 2013) (see Fig. 6). This has also been observed in humans, with increased bacterial endotoxin and 16S RNA after alcohol (Bala et al. 2014). The peripheral inflammatory response can then impact the brain and behavior through several routes (Critchley and Harrison 2013; Miller and Raison 2016). Systemic changes in cytokines, such as IL-1β, activate IL-1 receptors located on the vagus nerve (Ek et al. 1998). Vagal activation is transmitted to neural centers within the brain to promote the induction of sickness behaviors. Indeed, vagotomy reduces the sickness behavioral response to a peripheral injection of LPS, the TLR4 agonist (Bluthe et al. 1994). Secondly, peripheral cytokines can influence brain and behavior through transport in the blood. Proinflammatory cytokines in the blood can diffuse through permeable regions of the blood-brain barrier (BBB), such as the circumventricular organs, or be transported across the blood-brain barrier (Banks and Erickson 2010; Qin et al. 2007). TNFα transporters on the BBB are essential for systemic inflammation to cause brain inflammation (Qin et al. 2007). Similarly, in humans, circulating TNFα alters availability of the brain serotonin transporter (Krishnadas et al. 2016) and activation of immune signaling following vaccination causes confusion, fatigue, and loss of motivation (Harrison et al. 2009b). Fatigue following experimental inflammation in humans induces altered cortical metabolism and microstructure of the insular cortex (Harrison et al. 2015). Activated immune cells such as monocytes can be trafficked into the brain; however, this mechanism has been questioned with recent discoveries on microglial origination (Ginhoux et al. 2013). Once activated in brain, the innate immune response persists (Qin et al. 2008), thereby contributing to the neurobiology of addiction through the induction of alcohol consumptive behaviors as well as cognitive and emotive dysfunctions.

## Innate immunity may contribute to progressive stages of addiction

Current theories conceptualize addiction as encompassing a three-stage cycle that includes (1) binge/intoxication, (2) withdrawal/negative affect, and (3) preoccupation/craving (Koob and Volkow 2010) that contribute to the maintenance of substance use disorders. Accumulating evidence suggests that the innate immune system plays a role in each stage of the addiction cycle. With regards to the binge/intoxication stage, blockade of cocaine-induced upregulation of IL-1β in the ventral tegmental area (VTA) with the (+)-isomer of naloxone, a TLR4 antagonist, prevented the subsequent increase of dopamine release in the nucleus accumbens and diminished cocaine self-administration (Northcutt et al. 2015). Interestingly, this effect may be mediated in part by TLR4 signaling in microglia as treatment with minocycline, a microglial inhibitor, blocked cocaine-induced, conditioned place preference, supporting a role for the innate immune system in the rewarding effects of drugs of abuse. Similarly, self-administration of ethanol in the drinking in the dark paradigm in mice has been shown to increase expression of IL-1β in the basolateral amygdala. Injections of IL-1-ra, an IL-1 receptor antagonist, into the basolateral amygdala reduced ethanol self-administration in these animals (Marshall et al. 2016). Further, delivery of siRNAs for TLR4 or MCP-1 into either the VTA or central nucleus of the amygdala blunted ethanol self-administration in alcohol-preferring rats (June et al. 2015). Across multiple ethanol-drinking paradigms, transgenic mice with deletion of a single immune gene, such as CD14 and IL-6, consumed significantly less ethanol than matched wild-type controls (Blednov et al. 2005a, 2012). Conversely, stimulation of the innate immune system has been found to elicit ethanol consumptive behaviors. Indeed, systemic administration of LPS to adult mice produced a delayed but long-lasting increase in ethanol self-administration in mice (Blednov et al. 2011). Similarly, ventricular infusions of MCP-1 increase ethanol self-administration in rats (Valenta and Gonzales 2016). Together, these data provide evidence that the innate immune system, including TLRs, is involved in the binge/intoxication stage of addiction.

There is also evidence implicating innate immune activation in the withdrawal/negative affect stage of addiction. Substance abuse is associated with the development of negative affective states, such as anxiety and dysphoria (Koob and Le Moal 2005), which are commonly observed during the withdrawal stage of addiction. Indeed, the negative states associated with withdrawal are likely a driving force behind continued substance use, thereby contributing to cycles of abuse that might lead to dependence. Evidence indicates that it is during ethanol withdrawal that proinflammatory cytokine induction increases in brain. Freeman et al. (2012) found that expressions of MCP-1, NOS-2, TNFα, and the TNFα receptor are increased in the central nucleus of the amygdala following 48 h of withdrawal. Administration of LPS and cytokines sensitize the anxiety- and depressive-like behaviors associated with ethanol withdrawal (Breese et al. 2008). Knockout of TLR4 protects against ethanol withdrawal associated anxiety-like behavior and memory impairment (Pascual et al. 2011). These data implicate TLR4 and the innate immune system in ethanol withdrawal and the development of negative affect, although the mechanism remains to be fully determined. A probable point of convergence of withdrawal-induced innate immune upregulation and negative affect is hippocampal neurogenesis. Hippocampal neurogenesis is involved in regulation of mood and affective states (Malberg et al. 2000), and ethanol and LPS treatments reduce neurogenesis (see, e.g., Vetreno and Crews 2015). Importantly, reductions in hippocampal neurogenesis are observed during ethanol withdrawal that was associated with increased depression-like behavior on the forced swim test in mice (Stevenson et al. 2009). Further, the effects of ethanol withdrawal were prevented by anti-depressant treatment.

Stress causes induction of innate immune signaling in brain and mounting evidence indicates that immune activation can lead to alterations in affective states. As mentioned in the previous section, stress sensitizes microglia to inflammation in an HMGB1-dependent manner (Weber et al. 2015), and chronic stress activates microglia in multiple brain regions (Tynan et al. 2010) and causes depression-like behavior. Administration of microglial-modulating agents can block the development of depression-like behavior in rodents (Kreisel et al. 2014). Similarly, human studies also provide support for the involvement of stress and inflammation in affective disorders. Patients receiving systemic interferon-α for the treatment of hepatitis C can develop depression-like symptoms in the absence of a prior history of depression (Bonaccorso et al. 2002). Individuals with increased plasma levels of C-reactive protein (CRP), an acute phase marker of inflammation, are at greater risk for the development of depression (Young et al. 2014), and increased plasma level of CRP in adolescents is predictive of addiction later in life (Costello et al. 2013). Further, peripheral inflammation, which can increase innate immune signaling in brain, has been found to contribute to negative affect (Harrison et al. 2014). Human PET neuroimaging studies also find that expression levels of translocator protein (18 kDa), a marker of reactive microglia, are increased in the prefrontal cortex, insula, and anterior cingulate cortex of individuals during a major depressive episode (Setiawan et al. 2015). In depressed individuals who commit suicide, expression levels of microglial markers (i.e., Iba-1 and CD45) and the proinflammatory cytokine MCP-1 are increased in the cingulate cortex (Torres-Platas et al. 2014). These studies suggest that systemic inflammation leads to increased innate immune signaling in brain, contributing to the development of affective disorders.

Evidence supports an involvement of the innate immune system in affective disorders, but the mechanism remains to be elucidated fully. The literature provides evidence that a potential mechanism for innate immune involvement in the development of affective disorders involves alterations in the activity of neural circuitry that modulates affect. Administration of a typhoid vaccine was found to increase circulating levels of the proinflammatory cytokine IL-6 and significantly reduce mood (Harrison et al. 2009a). Interestingly, functional magnetic resonance imaging in these same participants revealed that the inflammation-induced reduction in mood was correlated with increased activity in the subgenual anterior cingulate cortex, a region implicated in the etiology of depression, and reduced connectivity of this region with the amygdala, prefrontal cortex, and nucleus accumbens (Harrison et al. 2009a). Studies also find that a variety of cytokine receptors, such as those for TNFα, IL-1β, IL-6, and the interferons, are expressed on neurons (Khairova et al. 2009), suggesting that cytokines act directly on neurons to influence their activity. Indeed, innate immune induction can reduce populations of serotonin (5-HT)-producing neurons in the raphe nucleus, and the serotonergic system has also been implicated in the etiology of affective disorders (Kelley et al. 2011). We found that AIE treatment persistently reduces populations of 5-HT-immunopositive neurons in the adult raphe nucleus, an effect that was mimicked by treatment with the TLR4 agonist LPS. Inflammation also increases activity of indolamine 2,3-dioxygenase (IDO), an enzyme that converts tryptophan (a precursor of 5-HT) to kynurenine, thereby reducing the synthesis of 5-HT (Wichers and Maes 2004). Interestingly, kynurenine has also been shown to induce depression-like behavior, and pharmacological inhibition of IDO blocks microglial activation and the development of depression-like behavior (O’Connor et al. 2009). Induction of innate immunity can also disrupt hippocampal neurogenesis, the process whereby new neurons are continuously added to the existing hippocampal neurocircuitry. Although heavily implicated in learning and memory processes, neurogenesis is also involved in mood and affective states (Malberg et al. 2000). Further, the ability of anti-depressants to alleviate depression-like behavior appears to be dependent upon hippocampal neurogenesis (Santarelli et al. 2003). Both inflammation (Ryan and Nolan 2016) and chronic stress (Kreisel et al. 2014) reduce neurogenesis and cause depression-like behavior. Further, stress induces IL-1β in the hippocampus, which decreases neurogenesis and contributes to depression, while inhibition of IL-1β blocks stress-induced decreases in neurogenesis and depression-like behavior (Koo and Duman 2008). While these data provide compelling evidence that the innate immune system modulates affect, the mechanism likely involves alterations to discrete nuclei as well as modifications to connectivity between regions. Together, these data implicate the TLR/innate immune system in the withdrawal/negative affect stage of addiction.

Evidence also implicates the TLR-innate immune system in the third stage of addiction involving preoccupation with drug taking and craving, which is associated with a heightened likelihood of relapse. Plasma levels of the proinflammatory cytokines IL-8 and IL-1β were positively correlated with indices of alcohol craving in human alcohol-dependent subjects (Leclercq et al. 2014). As mentioned above, stress (Madrigal et al. 2002, 2003) and drugs of abuse (Ang et al. 2001b; Crews and Vetreno 2014, 2016; Vetreno and Crews 2014) activate and sensitize (Frank et al. 2007; Qin and Crews 2012a) the innate immune system in brain. The innate immune system modulates alcohol consumptive behaviors (Blednov et al. 2005b, 2011; 2012; Liu et al. 2011b), and stressful events during abstinence might interact with the primed innate immune system leading to the craving to consume alcohol and other drugs of abuse. Treatment of abstinent human alcoholics with naltrexone, a TLR4 antagonist, reduced subjective reporting of craving (Nava et al. 2006). Similarly, treatment with the (+)-isomer of naltrexone, which is inactive at the opioid receptor, blocked heroin-seeking behavior in dependent rats (Theberge et al. 2013). Thus, TLR4 and innate immune signaling molecules contribute to the preoccupation/craving stage of addiction. Taken together, the evidence suggests that activation of the innate immune system in brain contributes to each stage of addiction.

## Summary

The discovery that innate immune mechanisms contribute to the neurobiology of addiction provides a novel approach for the treatment of alcohol use disorders. While innate immune activation contributes to alcohol consumptive behaviors, it also contributes to alcohol-induced neurodegeneration (Crews et al. 2004, 2006b; Obernier et al. 2002a, b; Qin et al. 2013). It is not clear how innate immune signaling alters specific neurocircuits. Innate immune signaling involves positive loops since cytokine and TLRs converge on NF-κB inducing additional cytokines and their receptors. However, HMGB1 and TLR activation could be the initial key signaling event. The current studies find innate immune signaling in most brain diseases with little evidence distinguishing activation in mental diseases and neurodegenerative diseases (e.g., addiction, Alzheimer’s disease, and depression). It remains to be determined whether therapies that target the innate immune system would be of benefit for the prevention of the progression to addiction and if these therapies could improve recovery from alcohol use disorders. Some studies have found that systemic anti-inflammatory agents reduce symptoms, but little is known on how brain inflammatory gene expression is impacted. Indeed, neurodegeneration would likely not be reversed, although interventions might allow for the recovery of normal synaptic function. Minocycline, a tetracycline antibiotic and microglial inhibitor (Plane et al. 2010), prevents ethanol induced-microglial activation and reduces alcohol self-administration (Agrawal et al. 2011; Qin and Crews 2012a). While studies are currently underway, further research is clearly warranted to explore the therapeutic potential of immune interventions in the treatment of alcohol use disorders.
